# An Exploratory Bibliometric Analysis of African Pharmacovigilance Research Output Using SCOPUS

**DOI:** 10.7759/cureus.56295

**Published:** 2024-03-16

**Authors:** Josiah T Masuka

**Affiliations:** 1 Dermatology, Faculty of Health Sciences, University of Zimbabwe, Harare, ZWE

**Keywords:** scientometrics, pharmacovigilance, drug safety, bibliometric analysis, africa

## Abstract

Most global pharmacovigilance (PV) data is derived from developed countries. However, the determinants of the differences in PV research output between developing and developed countries contributing to this discrepancy still need to be explored. The objective of the current study is to describe the publication trends and characteristics of pharmacovigilance-related research stemming out of Africa in comparison to that emanating from developed countries. A bibliometric analysis was carried out using the SCOPUS literature index for published global pharmacovigilance-related articles or documents pre-COVID-19. Data on annual publication trends, citations, author affiliations, and other study characteristics such as study funding were extracted and descriptively analyzed. Author co-citation and keyword co-occurrence analyses were also conducted and presented using VOSviewer software program version 1.6.15 (CWTS, Universiteit Leiden, Netherlands). During the period under review, a total of 27,516 documents were retrieved globally. Out of these, 588 (2.1 %), 9,438 (34.3 %), and 17,829 (64.8 %) were from Africa, Europe, and the International Council for Harmonisation of Technical Requirements for Pharmaceuticals for Human Use (ICH) founder member countries respectively. Annual publications have steadily increased, but at a slower rate in Africa compared to Europe. The mean annual publications and number of citations are significantly lower in Africa compared to Europe, p < 0.0001 for both parameters. The top 10 funders of African PV activities are European and American organizations. In conclusion, improved PV activity driven by international funders has been notable on the African continent. However, there is an increased need for local funding, government involvement, and legislation to improve PV activities on the African continent.

## Introduction and background

Pharmacovigilance (PV) is defined as “the science and activities relating to the detection, assessment, understanding, and prevention of adverse effects or any other drug-related problem” [1]. It aims to continuously update and enhance the available knowledge on any medicine’s safety and risk-benefit profile to improve patient care [2]. PV is aided by utilizing post-marketing adverse drug reaction (ADR) surveillance to detect unknown, rare and/or insufficiently described drug-related risks [2]. This safety information attempts to reflect real-world drug safety data as opposed to the clinical trial-derived ADR data which is limited by a short duration of exposure, and the controlled clinical trial conditions [3,4]. Active and passive ADR surveillance systems and the underpinning regulations help to increase the chances of timeous ADR detection [5]. This also enhances the available information to provide meaningful and more accurate risk-benefit analyses for any medicine, thereby improving patient care.

Currently, the majority of individual case safety reports (ICSRs) submitted to the World Health Organization’s (WHO) global ADR database, VigiBase® are derived from high-income countries [6]. Consequently, there is limited information on the burden of ADRs, the scope, patterns, characteristics, and performance of PV surveillance schemes in resource-limited countries. This is evidenced by the mere 0.8% contribution of ADRs submitted by African countries to VigiBase® [7]. Furthermore, there is little effort in most developing countries to collate, aggregate, and analyze ADR data as PV is not ordinarily considered a health priority in these settings [4,8]. At best, pharmacovigilance remains rudimentary [4] and predominantly a regulatory activity characterized by data collection and onward submission of ICSRs to VigiBase® in most African countries. There is limited government financial support which subsequently leads to an over-reliance on spontaneous reporting systems (SRS) with minimal active ADR surveillance systems [9,10]. However, in a few African Program for International Drug Monitoring (PIDM) member countries, PV has evolved to involve ADR data aggregation, analysis, dissemination, and integration into national policy [11]. 

The discrepancies in PV activities among African countries [11] and between African countries and most of the developed countries [6,7] need to be explored. They potentially result from the poorly funded and weakly regulated African PV systems [8]. It has been highlighted previously that African governments often provide initial political and technical support for the country to gain PIDM membership. Once attained, their financial support slows down with the resultant fragmentation of the PV systems [9]. Ampadu et al. showed the dismal performance of African PV surveillance systems after gaining PIDM membership as reflected by their meager ICSR reporting to VigiBase® [7]. The determinants of these observations still await further elucidation.

To address this information gap, we carried out a bibliometric analysis of drug safety literature [12] to describe publication trends, author affiliations, top publishing institutions and countries, and the themes of published articles stemming out of Africa pre-COVID-19. We also aimed to compare African publication characteristics - trends over time, annual publication rates, top journals, and citation characteristics to European and/or International Council for Harmonisation of Technical Requirements for Pharmaceuticals for Human Use (ICH) founder countries.

## Review

Methods

Study Design and Data Retrieval

The bibliometric analysis was done using Scopus, the largest curated, source-neutral abstract and citation database containing up to 24,600 active journal titles from 5,000 international publishers [13]. The publication data was retrieved from SCOPUS on 10/05/2020. Two different searches were done and later combined to yield the final result for each query on PV data from Africa and Europe respectively. The article title was searched using the “article title” tab for the following PV terms: “pharmacovigilance”, “drug safety”, “vaccine safety”, “adverse drug reaction”, “surveillance”, “adverse events”, “medication error”, “vaccine safety”, “medicine safety” and “signal”. This search was then combined with each country’s research output searched using the affiliation country” tab. Quotation marks and asterisks were used in the search strategy to enhance the accuracy and comprehensiveness of the results [14]. All document types were included in the statistical analysis of the retrieved data from inception to the end of the year 2019. All documents from 2020 were excluded as the retrieved documents comprise only a partial contribution in the year.

Bibliometric Analysis

The “Analyze search results” function in Scopus was used to provide the following statistics for the retrieved published documents: The study characteristics’ information such as the population - pediatric or adult; study design - database or non-database and/or active surveillance or passive surveillance was retrieved from each individual retrieved document and entered into an MS Excel spreadsheet for further analysis. The freely available, VOSviewer software program version 1.6.15 (CWTS, Universiteit Leiden, Netherlands) was used to produce network visualization maps of co-authorship by country and to analyze the most frequently occurring keywords [15,16]. The thickness of connecting lines was used as a measure of the strength of co-authorship and collaboration between countries [17]. 

Statistical Analysis

Descriptive statistics for measures of central tendency were displayed as means with their corresponding standard deviations. The independent t-test was used to test for statistically significant differences for chosen continuous variables. All statistical tests were done using the Medcalc® online statistical calculator [18] at the 5% significance level.

Ethical considerations

No ethical clearance was sought as this is a database study with no patient-identifying data and minimal risk.

Results

A total of 27,516 documents were retrieved globally by searching the titles of published documents using the defined search terms. Out of these, 588 (2.1 %), 9,438 (34.3 %), and 17,829 (64.8 %) were from Africa, Europe, and the ICH founding regulatory member countries (Europe, Japan, and the USA) [19] Globally, the top five PV publishing countries were the United States of America 8,554 (31.1 %), the United Kingdom 2,416 (8.8 %), France 1,873 (6.8 %), Germany 1,667 (6.1 %) and Canada 1,315 (4.8 %). In Africa, the top five PV publishing countries were South Africa 164 (27.9 %), Nigeria 91 (15.5 %), Egypt 58 (9.9 %), Ghana 46 (7.8 %), and Uganda 40 (6.8 %) respectively.

Description of Publication Trends

**Figure 1:**
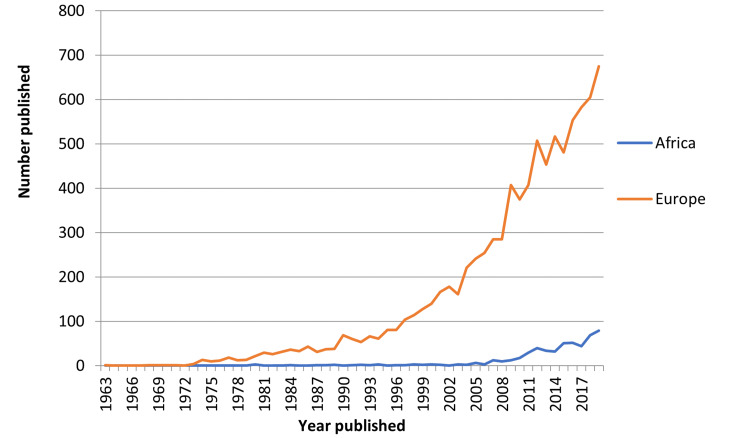
African vs European pharmacovigilance publication trends over the years

**Figure 2:**
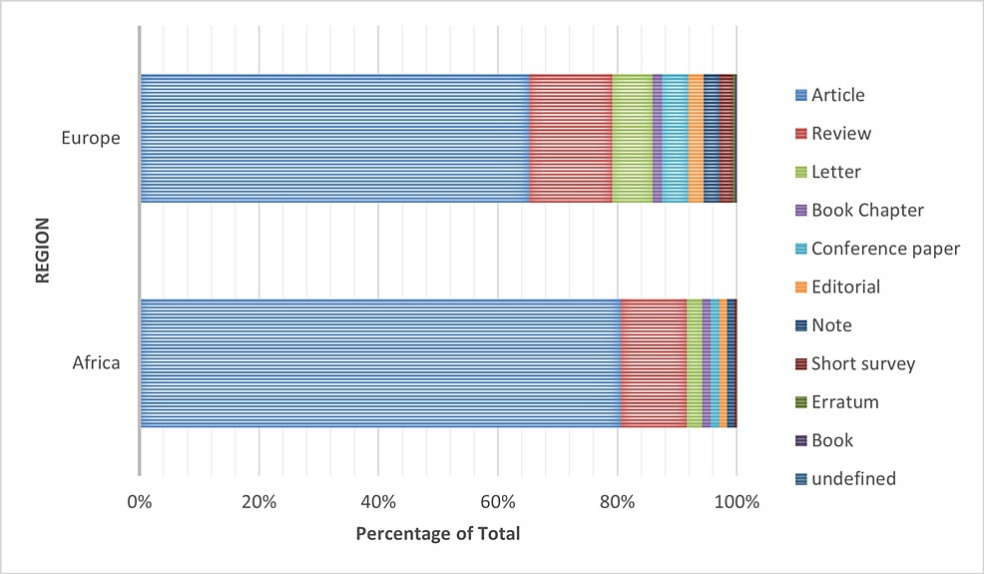
Retrieved published pharmacovigilance document types

Before 1975 and 1980 there were no PV publications for Europe and Africa respectively. However, the number of documents published annually has been increasing steadily over the years for both Africa and Europe. European growth has been steeper than the African counterpart from around 1996 as shown in Figure 1. Most of the retrieved documents were articles for both Africa and Europe as shown in Figure 2.

Publication Citation Characteristics

In general, developed countries publish significantly higher numbers of pharmacovigilance-related articles/documents as highlighted in Table 1. Furthermore, published articles/documents from developed countries have a higher impact as evidenced by the higher mean, mode, and median citations indices compared to African publications.

**Table 1:** Pharmacovigilance publication and citation characteristics PDS: Pharmacoepidemiology and Drug Safety; EuJCP: Europe Journal of Clinical Pathology; BJCP: British Journal of Clinical Pharmacology.

Characteristic		Africa	Europe	p value
Yearly publications	mean	9.55 ± 2.422	155.41 ± 25.243	<0.0001
Citations	total	6,315	175,836	
	mean	10.70 ± 1.469	25.09 ± 0.872	<0.0001
Open access/other	proportion (%)	278/310 (89.7%)	2,199/7,239 (30.4%)	<0.0001
Top 5 journals (number)	1	Drug Safety (31)	Drug Safety (391)	
	2	Plos One (31)	Therapie (321)	
	3	Vaccine (17)	PDS (210)	
	4	Pan Afr Med J (16)	EuJCP (186)	
	5	Malaria Journal (14)	BJCP (173)	

Author Keyword Analysis

A total of 1,100 different keywords were observed in the documents retrieved for PV in Africa over the study period. Of these keywords, 885 (80.5%), 117 (10.6%), and 40 (3.6%) appeared only once, twice, or thrice respectively. The most commonly occurring author keywords were adverse drug reaction(s) 81 (7.4%), pharmacovigilance 78 (7.1%), adverse events 57 (5.2%), medication errors 23 (2.1 %), and drug safety 17 (1.5 %). Vaccine safety is only mentioned in nine (0.8%) articles/documents.

Study Funding and the Institutions

Most African publications or documents did not indicate the study’s source of funding. In contrast to European-derived documents, most of the African-funded documents indicate foreign public entity or foundation funding with minimal local government funding. European PV publications were funded mostly by the pharmaceutical industry, national government, and/or foundation agencies as indicated in Table 2. The most active PV publishing institutions are also indicated in Table 3, showing a predominance of university-driven activity both in Africa and Europe. Notable international author collaborations were observed for Ghana, Nigeria, and South Africa-based authors, particularly with other African-based authors and European-based authors.

**Table 2:** Published pharmacovigilance study funding characteristics USAID: United States Agency for International Development; NIAID: National Institute of Allergy and Infectious Diseases; NIHR: National Institute for Health and Care Research; AHRQ: Agency for Healthcare Research and Quality.

Position	Africa	N	Europe	N	ICH founders	N	Global	N
1	National Institutes of Health	14	Pfizer	102	National Institutes of Health	394	National Institutes of Health	409
2	World Health Organisation	13	Seventh Framework Programme	75	Pfizer	163	Pfizer	165
3	Centers for Disease Control and Prevention	12	Medical Research Council	66	National Cancer Institute	141	National Cancer Institute	152
4	Medical Research Council	12	Norvatis	62	Japan Society for the Promotion of Science	111	National Natural Science Foundation of China	149
5	Bill & Melinda	11	National Institutes of Health	58	Centers for Disease Control and Prevention	102	Japan Society for the Promotion of Science	112
6	NIAID	8	NIHR	56	Novartis	95	Centers for Disease Control and Prevention	107
7	USAID	6	Bristol-Myers Squibb	51	Bristol-Myers Squibb	94	Norvatis	105
8	Pfizer	5	AstraZeneca	48	AHRQ	90	Bristol-Myers Squibb	98
9	Wellcome Trust	5	European Commission	43	National Heart, Lung and Blood Institute	90	AHRQ	94
10	Forgaty	4	Roche	43	NIAID	84	National Heart, Lung and Blood Institute	91

**Table 3:** Most active pharmacovigilance publishing institutions AP-HP Assistance Publique: Assistance Publique–Hôpitaux de Paris; CHU de Toulouse: Centre Hospitalier Universitaire de Toulouse; Erasmus MC: Erasmus University Medical Center.

Position	Africa	N	Europe	N	ICH founders	N	Global	N
1	University of Cape Town	56	Inserm	406	Food and Drug Administration	489	Food and Drug Administration	496
2	World Health Organisation	42	AP-HP Assistance Publique	222	Harvard Medical School	473	Harvard Medical School	482
3	University of KwaZulu-Natal	32	CHU de Toulouse	194	Inserm	406	Inserm	408
4	University of Ghana	31	Erasmus MC	183	Centers for Disease Control and Prevention	365	Centers for Disease Control and Prevention	369
5	Centers for Disease Control and Prevention	30	Faculte de Medecine Universite Paul Sabatier	153	Bringham and Women’s Hospital	294	University of Toronto	329
6	Makerere University	24	University of Liverpool	147	Institute for Safe Medication Practices	263	Bringham and Women’s Hospital	297
7	Cairo University	17	Universite de Bordeaux	146	AP-HP Assistance Publique	222	Institute for Safe Medication Practices	266
8	University of Lagos College of Medicine	17	Uppsala Monitoring Centre	141	CHU de Toulouse	194	AP-HP Assistance Publique	223
9	University of Lagos	16	Karolinska	123	Massachusetts General Hospital	189	CHU de Toulouse	194
10	University of Witwatersrand	16	Universite de Paris	123	Erasmus MC	183	Massachusetts General Hospital	189

Discussion

In this study, we sought to describe the publication trends and characteristics of pharmacovigilance-related research stemming out of Africa in comparison to that from developed countries. This study indicates that pharmacovigilance literature output is steadily increasing annually in keeping with trends observed for public health research output from Africa [20] and global trends [21,22], but at a slower rate compared to Europe. Most of the African pharmacovigilance research output is funded by European and American funders through international public health initiatives. It mostly occurs in collaboration with authors from these countries as well [20]. Similar to previous findings, the most frequent author keywords were adverse drug reaction(s) and pharmacovigilance globally [21]. However, themes such as signal detection analysis and disproportionate analysis were observed in literature from developed countries. In addition, medication errors, lack of efficacy, quality, and counterfeit drug issues are largely not presented in the available literature as expected from fully functional PV systems [4].

Unlike the ICH founding member countries, African PV systems are weak, inefficient, and lack the appropriate infrastructure, funding, government support, and legal framework to conduct efficient PV activities [4,10,23]. The funding for the majority of PV activities is indirectly provided through other public health initiatives for diseases such as HIV/AIDS, tuberculosis, and malaria [23]. This possibly explains the small extent of African PV schemes in terms of therapeutic areas covered, the number of ICSRs submitted to VigiBase®, and the scope of PV activities [7]. Isah et al. found that in addition to the significant donor funding, most African PV systems lack independence from regulatory agencies regarding operational and fiscal governance, limiting their operability [24]. The observed 2.1% African literature output mirrors the mere 0.88% African ICSR contribution to the global ADR database, VigiBase® [7] indicating the lack of investment in the field. This is possibly related to the lack of PV prioritization on the continent and the over-reliance on funding from international development partners to conduct PV activities [25] as also indicated in this study.

Several issues need to be addressed to redress the observed PV limitations in Africa. Firstly, firm regulatory policy and enforceable PV legislation need to be adopted to ensure adequate funding and authority to the local regulatory authorities (RAs) [24,26]. RAs and especially marketing authorization holders (MAHs) should be legally accountable for the provision of risk management plans for any registered medicine in Africa as occurs in ICH territories. This may enforce RAs and MAHs to fund relevant PV activities in Africa. Secondly, as occurs in Europe, pharmacovigilance guidelines should be developed to define the scope and practice of the discipline and assist in the inclusion of quality and counterfeit drug-related safety issues. Thirdly, raising PV awareness through public engagement and increasing healthcare practitioner PV education during basic professional training may increase the reach and extent of the discipline [26]. Lastly, the adoption of electronic health information systems and artificial intelligence together with the implementation of joint, regional pharmacovigilance programs could potentially improve and sustain the continent’s pharmacovigilance practices [22,27,28]. 

This study has several limitations inherent to the study design and the search strategy adopted by the researchers as previously discussed in other bibliometric analyses [17,29]. Firstly, this study omitted non-SCOPUS-indexed publications, thereby limiting the number of potential data-contributing articles. This could have unequally impacted African publications as most African research is not indexed outside the continent [30] thus potentially providing erroneous conclusions. Secondly, false positives may have been included as it is difficult to distinguish articles that focus on pharmacovigilance from those that tangentially mention the term [29]. However, SCOPUS is the largest indexing database, thus a representative sample may have been analyzed in the current study. Despite these limitations, to the best of our knowledge, this study is the first to analyze bibliometric indicators of pharmacovigilance in Africa and still potentially provides comprehensive data on the continent’s productivity in pharmacovigilance research. Further studies are required to explore and compare the types of data utilized, study designs, and the populations explored in Africa versus the developed countries. Some populations such as pediatric patients may still be under-represented given the lack of enabling legislation in Africa.

## Conclusions

African PV systems still need to meet the WHO’s prescribed bare minimum of collecting, collating, and ADR risk management. These PV systems are not fully functional as yet. Proactive, risk proportionate, and patient-centered public health approaches will need to be gradually introduced to make African PV schemes robust enough to meet local needs for benefit-risk balance analyses as occurs in Europe. This is especially important given the improved access to medicines on the continent necessitating strong and effective health systems including drug monitoring systems. Increased local funding, government involvement, and legislation may potentially improve Africa’s PV output - ADR reports, publications, and ultimately risk minimization activities.
